# A 20-week, randomized controlled trial to determine the efficacy of the Aging Well through Interaction and Scientific Education – Action Plan (AgeWISE-AP) program in older Veterans

**DOI:** 10.1186/s12877-025-06207-x

**Published:** 2025-08-04

**Authors:** Maureen K. O’Connor, Julianne Szemko, Jaye E. McLaren, Lauren R. Moo, Emily E. Metcalf, Andrew Nguyen, Malissa L. Kraft, Brandon Frank, David Salat, John J. Randolph, Yorghos Tripodis

**Affiliations:** 1 Neuropsychology Service, Bedford VA Healthcare System, 200 Springs Rd, Bedford, MA 01730 USA; 2 Geriatric Research Education and Clinical Center, Bedford VA Healthcare System, Bedford, MA USA; 3 Department of Neurology, Boston University, Boston, MA USA; 4 Department of Psychiatry, Geisel School of Medicine, Dartmouth College, Hanover, NH USA; 5 School of Public Health, Boston University, Boston, MA USA; 6 Department of Neurology, Harvard Medical School, Boston, MA USA; 7 Center for Translational Cognitive Neuroscience, Boston VA Healthcare System, Boston, MA USA

**Keywords:** Aging, Lifestyle factors, Older adult, Veterans

## Abstract

**Background:**

Advancing age is the number one risk factor for cognitive decline and dementia, making cognitive aging a concern for the nearly half of Veterans who are age 65 and older. There has been a growing appreciation for the value of nonpharmacological lifestyle interventions in dementia risk reduction, including consistently exercising, eating a healthy diet, getting sufficient sleep, socializing, and engaging in cognitively stimulating activities. The 12-week Aging Well through Interaction and Scientific Education program (AgeWISE) provides Veterans with education about brain aging and modifiable lifestyle factors that promote cognitive resilience. A pilot study conducted in 2013 revealed modest improvements in AgeWISE participants’ perceived ability to control brain aging.

**Methods:**

The recently funded AgeWISE-Action Plan (AgeWISE-AP) aims to capitalize on the increased feelings of control over brain aging found in the AgeWISE program through the addition of an action plan (AP) component. The AP involves 8 individualized sessions with a Brain Health Interventionist in addition to AgeWISE group participation. A total of 128 Veterans will be enrolled in the study and each will be randomized to one of the two study arms: AgeWISE-AP (intervention arm) or a no treatment control condition. Outcome measures will be administered at baseline (pre-intervention), immediately post-intervention, and 3- and 6-months post-intervention. These outcome measures assess engagement in brain-healthy lifestyle factors, psychological well-being, and cognition. Linear mixed regression models will be used to test the effect of AgeWISE-AP. An exploratory outcome will focus on structural imaging data acquired from a subset of participants at baseline and one year to investigate potential volumetric changes in brain regions of interest.

**Discussion:**

Multi-component interventions that provide brain aging education, improve feelings of control over brain aging, and deliver practical assistance with goal setting and attainment are critically needed to improve cognitive outcomes for older adults. The AgeWISE-AP program aims to increase engagement in protective lifestyle factors, and directly target and improve psychological wellbeing and cognition in older Veterans.

**Trial registration:**

This study was registered with ClinicalTrials.gov (NCT06006962) on August 17, 2023.

## Background

### Introduction

The number of older Veterans is increasing. Compared to civilians, who have a median age of 44, Veterans’ median age is 64 [1]. Age is the number one risk factor for cognitive decline and dementia, making cognitive aging a concern for the nearly half of Veterans currently age 65 and older, as well as for the many Veterans that are approaching age 65. The prevalence of dementia in Veterans stands around 10% [2] and is expected to increase to 22% by 2033 [3]. Veterans are also more likely than non-Veterans to present with known psychological, physical, and social risks associated with dementia that make them particularly vulnerable to cognitive decline with aging [4]. For example, mental health conditions such as depression, anxiety, and alcohol abuse are prevalent in the Veteran population and increase the risk of cognitive decline and dementia [2, 4]. Post-Traumatic Stress Disorder (PTSD) in Veterans is also strongly associated with dementia, with some studies suggesting that a diagnosis of PTSD doubles the risk of developing dementia [5]. Traumatic brain injury, also prevalent among Veterans, increases dementia risk [6]. In less than 10 years, many of those Veterans who served in Iraq and Afghanistan, where traumatic brain injury was considered a signature wound, will turn 65 years old. Research has suggested that mild traumatic brain injury from military blast exposure accelerates brain aging [7]. In addition, cardiovascular health conditions, such as hypertension, dyslipidemia, diabetes, heart disease, and obesity are risk factors for cognitive decline and are more prevalent and tend to occur at earlier ages in Veterans compared to civilians [8, 9]. Social factors associated with cognitive decline, such as isolation and homelessness, are also more common in the Veteran population [10, 11]. Moreover, the percent of female Veterans and Veterans from diverse backgrounds [12], populations shown to be at increased risk for developing dementia compared to White males, is on the rise [13]. For these reasons, cognitive aging has been established as a Department of Veterans Affairs (VA) priority.

Evidence from the past two decades demonstrates the strong role nonpharmacological lifestyle interventions play in improving brain health. Consistent exercise, a healthy diet, sufficient sleep, socialization, and engagement in cognitively stimulating activities can reduce dementia risk [14]. In fact, several recent studies suggest that late-life cognitive changes are better explained by environmental influences than genetic factors [15]. Importantly, many of these environmental factors are modifiable through lifestyle changes. Numerous studies have found that regular exercise maintains cognitive health and prevents age-related cognitive decline [16–20] through both direct impact on brain structure and function [21] and indirect impact through the mitigation of physical and psychological conditions related to cognitive decline [22]. Diet al.so plays a central role in reducing cerebrovascular risk factors including high blood pressure, diabetes, high cholesterol, and obesity, that increase risk for developing dementia [23–26]. For example, a Mediterranean-style diet (MeDi) helps control cerebrovascular risk factors associated cognitive decline [27, 28] slows cognitive decline, and reduces the risk of dementia [29, 30]. Additionally, adequate sleep promotes healthy cognitive functioning, whereas reduced or excessive sleep leads to cognitive deficits [31]. Further, chronic sleep difficulties and sleep disorders increase the risk of dementia [32]. Socialization is also associated with better cognition [33]. Individuals that engage in regular social activity perform better on cognitive tests and have fewer cognitive complaints [34]. Having robust social networks buffers the impact of brain disease on functional activity [35]. Conversely, loneliness and isolation are associated with poorer cognition, accelerated cognitive decline, and increased risk of dementia [36]. Furthermore, engagement in cognitively stimulating activities, defined as leisure activities with intellectual demands, has been shown to improve cognition and reduce dementia risk [37, 38], with studies reporting a 33–36% reduced risk of dementia relative to those engaging in less activity [39–42]. Various brain biomarkers also help to support the effectiveness of lifestyle interventions. For example, numerous lifestyle activity studies and interventions have been supported by the addition of structural neuroimaging. In fact, older individuals who are more socially and cognitively engaged show greater global and regional gray matter volume and integrity [43, 44]. Greater adherence to a Mediterranean diet has been associated with less atrophy in areas linked to Alzheimer’s disease, including frontal, parietal, and medial temporal regions [45, 46]. Multiple studies have also documented increased white matter plasticity and greater brain volume in hippocampal, frontal, and cingulate cortex regions as a result of exercise-oriented lifestyle interventions [47–49]. In addition, structural neuroimaging can be used in lifestyle intervention studies at baseline to compare activity engagement and other participant characteristics for a better characterization of the study sample to help further identify characteristics of intervention responders.

### Pilot study

The 12-week Aging Well through Interaction and Scientific Education program (AgeWISE) was developed for aging Veterans in response to a recognized need to disseminate information about brain health and provide the most up-to-date scientific information about lifestyle factors associated with brain health. Aligned with the focus of the National Institutes of Neurological Disorders and Stroke, Mental Health, and Aging [50], the Centers for Disease Control and Prevention, and the Alzheimer’s Association [51], AgeWISE was created with three goals in mind: (1) to provide older Veterans with education about normal brain aging and brain diseases of aging; (2) to present older Veterans with the latest scientific literature on lifestyle factors related to healthy brain aging; and (3) to teach older Veterans techniques and skills that could help them manage some of the normal age-related changes in memory and thinking that impact everyday functioning. In 2013, a pilot study using a randomized controlled design was undertaken to determine feasibility and acceptability, and to conduct preliminary comparisons of the effect of the intervention to standard practice (a no treatment control condition; VA Rehabilitation Research & Development NCT02023944; see also: O’Connor et al., 2018 [52]). Individuals participating in AgeWISE felt the program improved their memory and thinking and provided them with new skills and useful information. They enjoyed the program and were likely to recommend the program to a friend. They particularly enjoyed learning about the role of lifestyle factors in healthy brain aging. Intervention participants also reported a subjective sense of increased control over their brain health and showed a quantitative increase in their sense of control over their brain health on formal measures.

### The present study

**Fig. 1:**
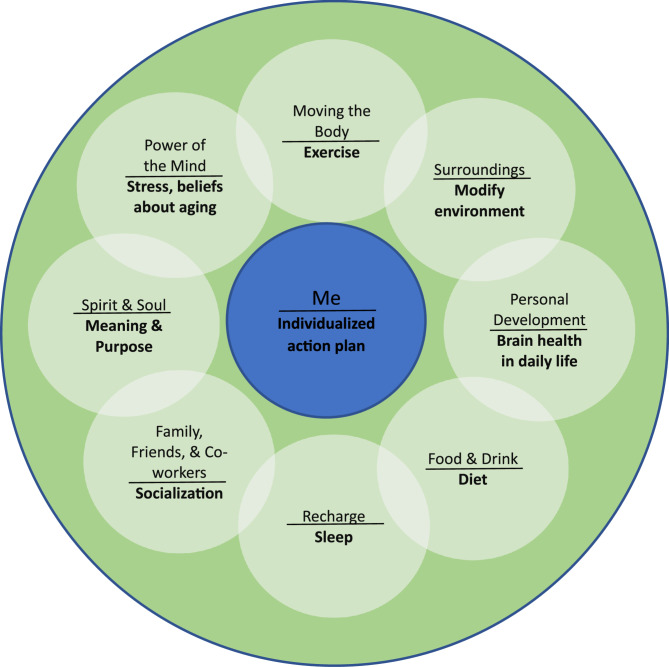
Circle of Health Self-Care Practices

As the VA faces increasing numbers of aging Veterans, investing in improving brain health with lifestyle-based programming has the opportunity to improve older Veterans’ quality of life, promote independence and aging in place, and reduce healthcare costs. The current program described here, referred to as AgeWISE-Action Plan (AgeWISE-AP), aims to capitalize on the initial pilot study findings by adding an individualized action plan (AP) component to the original AgeWISE group intervention. AgeWISE-AP begins with engagement in the psychoeducational AgeWISE group targeting each of the 8 self-care practices of the Circle of Health (see Fig. 1), beginning with emphasis on the relationships between exercise (Moving the Body), diet (Food & Drink), sleep (Recharge), social connection (Family, Friends, & Co-workers), and meaning and purpose (Spirit & Soul) to brain health. AgeWISE also provides information and instruction on techniques to reduce stress and negative beliefs about aging (Power of the Mind), incorporate brain healthy activities into daily life (Personal Development), and modify the physical environment to minimize the impact of cognitive changes (Surroundings). In week 9 of the group, Veterans begin additional engagement in the AP component. The AP component is designed to leverage AgeWISE group increases in feelings of control over brain health by engaging Veterans in a one-on-one, collaboratively created behavior modification plan through a partnership between the Veteran and a Brain Health Interventionist. The goal of the AP component is to increase Veteran engagement in brain healthy lifestyle activities. Each Veteran meets one-on-one with the Brain Health Interventionist to develop and execute a personal action plan driven by the Veteran’s own values, needs, and goals and centered in the Veterans Health Administration’s Whole Health System, which emphasizes Veteran-led care. The AP component begins in week 9 of the group, with Veterans adding an individual meeting with the Brain Health Interventionist to their week. During weeks 9–12 of group participation, Veterans are also meeting with the Brain Health Interventionist to develop their action plan. The Action Plan recognizes that each Veteran has unique values, goals, and priorities (the “me” represented in the center of the Circle of Health) related to their brain health.

## Methods

### Study aims

Participants in AgeWISE-AP will be compared to a no treatment control group. There are three primary aims of the present study as well as a fourth exploratory aim.

- Aim 1: To determine whether AgeWISE-AP increases older Veterans’ engagement in lifestyle factors that promote brain health. AgeWISE provides information about the differences between normal and diseased brain aging and lifestyle factors that contribute to brain health. The action plan (AP) component will use this foundation to collaboratively create an individualized brain health plan to increase Veteran engagement in brain healthy lifestyle activities. We hypothesize that AgeWISE-AP participants will demonstrate increased engagement in brain-healthy lifestyle activities compared to the control group.
- Aim 2: To determine whether AgeWISE-AP improves psychological wellness. AgeWISE provides information about the relationship between cognitive aging and affective states (e.g., depression) and attitudes about aging, and teaches stress reduction techniques. The action plan (AP) will provide additional individualized supports to improve psychological well-being with personalized goals and lifestyle modifications (e.g., diet, exercise, sleep, cognitive stimulation). We hypothesize that AgeWISE-AP participants will show increases in perception of control over cognitive aging, meaning and purpose in life, quality of life, and self-efficacy, as well as improved attitudes toward aging and decreased loneliness, depression, and anxiety compared to the control group.
- Aim 3: To determine whether AgeWISE-AP improves cognition. Cognitive strategies to improve cognition and functioning are presented and practiced over three AgeWISE sessions, with homework to improve generalization to day-to-day life. Engagement in brain-healthy lifestyle activities will be accomplished through the action plan (AP). We hypothesize that self-reported memory contentment, ability, and compensatory strategy use will increase. We also hypothesize that objective cognitive performance will be better for AgeWISE-AP participants compared to the control group.
- Exploratory Aim 4: To determine whether AgeWISE-AP influences biomarkers of brain health using structural neuroimaging methods. We hypothesize that at one-year follow-up, AgeWISE-AP participants will have less volumetric decline in brain regions of interest compared to the control group.

### Study overview

The current study is a Phase III randomized controlled clinical trial. The study will employ two arms: the AgeWISE-AP intervention arm and a no treatment control group arm.

#### AgeWISE-AP intervention arm

AgeWISE-AP includes engagement in the original 12-week, one hour per week AgeWISE group plus an additional 8-session individualized action plan. Group AgeWISE sessions include education, discussion, and in-class exercises designed to practice introduced skills. Out of session homework assignments are given weekly to promote generalization of training into daily life. Table 1 displays an outline and summary for the 12 sessions in the intervention (as seen in O’Connor et al., 2018 [52]). During week 9 of the AgeWISE group, Veterans will begin the novel AP component of the intervention.

**Table 1:** AgeWISE Group Session Descriptions

	AgeWISE Group Sessions
Week	Description of Content/Goals
1.	• Make introductions and begin building connections and peer support • Learn and practice two new cognitive strategies
2.	• Summarize brain functioning as it relates to normal aging • Quiz on beliefs about aging
3.	• Provide education about cognitive domains including memory, attention, executive functioning, language, and visuospatial/perceptual skills, and the impact cognitive aging has on these abilities
4.	• Provide education about dementia and the differences between normal age-related changes in cognition and dementia
5.	• Review impact of mood states such as depression, anxiety, and stress on cognitive functioning and discuss (and practice) techniques to manage stress • Discuss the impact that negative stereotypes about aging can have on successful aging and learn techniques to counter negative aging stereotypes
6.	• Discuss the contribution of exercise to successful aging and increase motivation to increase physical activity level • Present guidelines for exercise in older adults and provide specific examples of what constitutes exercise
7.	• Review lifestyle factors of diet, socialization, cognitive stimulation related to successful cognitive aging
8.	• Provide overview of normal age-related changes in attention and common memory failures associated with changes in attention • Review specific external and internal strategies designed to improve attention in daily life • Practice strategies in session
9.	• Present internal strategies to improve functioning • Practice strategies in session
10.	• Practice strategies taught in Sessions 8 and 9 • Provide time for troubleshooting and discussion of how each strategy can be utilized in day-to-day life
11.	• Present external strategies to improve functioning
12.	• Review previous session content, troubleshoot, and process the intervention experience

The AP component will be run by a doctoral level occupational therapist serving as the Brain Health Interventionist. The Brain Health Interventionist meets with Veterans individually, once a week over the first month (final 4 weeks of AgeWISE) to define at least three specific goals across lifestyle factors associated with dementia risk (exercise, diet, sleep, socialization, cognitive stimulation) and create an actionable plan to meet those goals. The Brain Health Interventionist then meets with the Veteran every other week (4 additional sessions for a total of 8 individual sessions) to monitor progress, identify barriers to progress, and revise the plan as needed. The Brain Health Interventionist uses the Whole Health Personal Health Inventory and Personal Health Plan worksheets, modified for the current study to focus on brain health, to help determine targeted goals for change. Motivational interviewing techniques are incorporated throughout individual sessions. The Brain Health Interventionist assists with coordination of referrals to existing programs within and outside of the VA system specific to Veteran-identified goals. For example, a Veteran who identifies increased exercise as a goal might be encouraged to join the VA GeroFit program or attend classes at their local YMCA. As needed, the Brain Health Interventionist may make the referral, provide the phone number for YMCA membership, print a copy of the class schedule, and help the Veteran brainstorm solutions to barriers such as transportation.

#### Control group arm

The control condition is a no treatment control. Although other control conditions could have been proposed, no treatment was chosen for this study for a number of reasons including (a) the desire to fully capture what occurs in the real lives of older Veterans, which typically involves no active treatment (minimal education and skills training related to cognitive aging concerns from other professionals may occur, as may self-generated acquisition of knowledge regarding cognitive aging), and (b) the desire to minimize the influence of increased socialization with other older Veterans having similar concerns, as we suspect that such interaction is one active ingredient of the intervention. Following study completion, control group Veterans will be invited to participate in the AP intervention as a clinical service.

### Participants

A total of 128 Veterans will be recruited into the study staggered across Years 1 and 2 to ensure that the workload is manageable (an average of 16 participants will be recruited per study quarter). Recruitment takes place at the Bedford VA Healthcare System. All potentially eligible Veterans are asked if they would like to hear information about a study related to cognitive aging. Those who agree are given a study flyer and provided with a brief description of the study and procedures. Those who express interest are screened for eligibility and will complete the informed consent process and HIPAA authorization (see Table 2 for a schedule of enrollment, interventions, and assessments). During the informed consent process, participants are given the option of undergoing brain imaging at baseline and one-year follow-up for Aim 4. Participants that agree to neuroimaging undergo an additional informed consent process during the first of two brain imaging sessions, and complete a screening questionnaire specific to brain imaging safety. The informed consent processes occur in a private office setting and the information is presented both verbally and in written format. Veterans are asked to paraphrase informed consent information to ensure adequate understanding. Veterans are given ample time to ask questions about the study. After the informed consent, Veterans undergo a study screening that includes administration of the Montreal Cognitive Assessment (MoCA). Inclusion criteria for the present study includes Veterans *≥* 60 years old with concerns about brain aging who want to learn more about cognitive aging; and English speaking as all intervention materials are written in English. Exclusion criteria for the present study includes Veterans with impairment on a cognitive screening measure, as determined using a MoCA cutoff score for dementia of < 24 [53], or self or other reported diagnosis of a brain disorder affecting cognition such as Alzheimer’s disease, Parkinson’s disease, other dementia, major stroke, brain injury, or diagnosis of psychotic disorder such as schizophrenia; and active alcohol or substance abuse.

**Table 2:** Schedule of Enrollment, Interventions, and Assessments

Timepoint	Study Period					
	Enrollment	Allocation	Study Intervention	Follow-up		
	-t_1_	0	20-weeks	Immediate	3-month	6-month
Enrollment						
Informed Consent	x					
Eligibility Screen	x					
Allocation		x				
Intervention						
AgeWise-AP			x			
No treatment Control			x			
Assessments						
MoCA	x					x
CHAMPS	x			x	x	x
SIG-QOL	x			x	x	x
HPLP-II	x			x	x	x
GSAQ	x			x	x	x
General and Memory Specific Control Beliefs Scale	x			x	x	x
PGCMS	x			x	x	x
NIH Toolbox Meaning and Purpose Short Form	x			x	x	x
PHQ-9	x			x	x	x
GAD-7	x			x	x	x
GSE	x			x	x	x
MMQ	x			x	x	x
Demographics	x			x	x	x
Optional Sub-Study						
MRI	x					x

### Procedures

After eligible Veterans complete informed consent, screening, and baseline measures for study inclusion, each is randomized to one of the two study arms: AgeWISE-AP (intervention arm) or the control condition. Randomization uses two computer-generated numbered lists; one for each condition. The lists are generated through a permuted block design. With this approach, each randomization list consists of two AgeWISE-AP assignments and two control condition assignments in random order. This approach not only guarantees balance in the two conditions, but also balances assignments due to the time of recruitment.

The manualized AgeWISE group classes are run by doctoral level psychologists within the Bedford VA Healthcare System’s Neuropsychology Department. The study team discusses any issues related to the intervention sessions during weekly meetings. The AP component is run by a doctoral level occupational therapist serving as the Brain Health Interventionist to increase older Veteran participation in lifestyle activities associated with healthy brain aging. The Brain Health Interventionist helps the Veteran complete the Whole Health Personal Health Inventory and Personal Health Plan worksheets, which were modified to focus on brain health, to determine personalized goals, create an actionable plan to meet goals, and provide assistance with program referrals as needed (4 initial weekly sessions, 1 h per week). The Brain Health Interventionist then meets with the individual Veteran every other week for the final 8 weeks (4 additional sessions, 1 h every other week, for a total of 8 AP sessions) to review goals, boost motivation through accountability check-ins, determine any barriers to behavior change, and modify and adjust goals accordingly.

The study has been approved by the VA’s Central Institutional Review Board (cIRB) and Scientific Review Committee. Participant recruitment is at a target rate of 1–2 Veterans a week, projected at approximately 16 Veterans per study quarter during months 4 through 27 (see Table 3). The Bedford VA Healthcare System is a Category 3 facility located in the north-west suburbs of Boston serving approximately 20,000 Veterans with the bulk of clinical services in mental health, primary care, and geriatrics and extended care. Participants will be recruited from neurological, psychological, geriatric, and primary care services.

**Table 3:** Project Timeline

Project Activity	Year 1				Year 2				Year 3				Year 4			
	Q1	Q2	Q3	Q4	Q1	Q2	Q3	Q4	Q1	Q2	Q3	Q4	Q1	Q2	Q3	Q4
Study start-up	x	x														
Interventionist training	x	x														
Participant recruitment		x	x	x	x	x	x	x	x							
Baseline data collection		x	x	x	x	x	x	x	x							
Baseline neuroimaging		x	x	x	x	x	x	x	x							
Intervention delivery			x	x	x	x	x	x	x	x	x					
Immediate data collection				x	x	x	x	x	x	x	x					
3-month post intervention					x	x	x	x	x	x	x	x				
6-month post intervention						x	x	x	x	x	x	x	x			
Follow-up neuroimaging						x	x	x	x	x	x	x	x			
Analyze data				x	x	x	x	x	x	x	x	x	x	x		
Manuscript preparation					x	x	x	x	x	x	x	x	x	x		
Grant preparation: Durability of Rehabilitation Interventions for Veterans (DRIVE) Award													x	x	x	x

For Aims 1 through 3 (Primary Aims), the baseline evaluation takes participants an average of 1.25 h (1 h and 15 min) to complete the following measures, including collection of the demographic data (see below); sessions can be broken into two sessions as needed to minimize any fatigue. Follow-up evaluations take place immediately after the AgeWISE-AP intervention has ended (immediate post intervention), 3-months post intervention, and 6-months post intervention (i.e., 1 year after enrollment) and take an estimated hour and a half to complete. Structural imaging data is collected at baseline and again at one year, taking an estimated hour and a half to complete, with 30–45 min preparation for scanning and another 45 min of time spent in the scanner.

### Assessments

The outcome measures for this study were chosen to inform our specific aims. We seek to investigate whether AgeWISE-AP boosts older Veterans’ engagement in lifestyle activities that promote brain health (Aim 1) and improves psychological wellbeing (Aim 2) and cognition (Aim 3) compared to a treatment as usual (no intervention) control group. We will additionally explore whether there is an intervention effect on biomarkers of brain health using structural neuroimaging methods (exploratory Aim 4). The following measures will be used to test the hypotheses associated with each aim. A detailed description of each measure is provided below. A demographic questionnaire will be administered only at baseline that captures basic data including age, gender, race, educational history, occupational history, medical history, and psychiatric history.

### Outcome measures

The following instruments will be administered at baseline (pre-intervention), immediately post intervention, and 3- and 6-months post intervention (see Table 4).

**Table 4:** Assessment Instruments

Instrument	Purpose	Admin Time
**Aim 1: Lifestyle Factors**		
Community Healthy Activities Model Program for Seniors Questionnaire (CHAMPS)	Engagement in physical, cognitive, & social activity	20
Single Item Global Quality of Life Scale (SIG-QOL)	Quality of Life	2
Health-Promoting Lifestyle Profile II (HPLP II)	Health responsibility, physical activity, nutrition, spiritual growth, interpersonal relations & stress management	7
Global Sleep Assessment Questionnaire (GSAQ)	Sleep	5
**Aim 2: Psychological Wellbeing**		
General and Memory Specific Control Beliefs Scale	Perceived control over cognitive health	4
Philadelphia Geriatric Center Morale Scale (PGCMS)	Attitudes toward aging, lonely dissatisfaction	2
NIH Toolbox Meaning and Purpose	Meaning and purpose in life	5
Patient Health Questionnaire 9 (PHQ-9)	Depression	5
Generalized Anxiety Disorder-7 (GAD-7)	Anxiety	5
Generalized Self-Efficacy Scale (GSE)	Self-efficacy	5
**Aim 3: Cognition**		
Multifactorial Memory Questionnaire (MMQ)	Self-reported memory contentment, memory ability, & strategy use	5
Montreal Cognitive Assessment (MoCA)	Objective cognitive performance	10

#### Aim 1: lifestyle factors

*Community Healthy Activities Model Program for Seniors Questionnaire* (CHAMPS) [54] is a 41-item scale that explores the frequency and duration of light, moderate, and vigorous physical activities assessed using weekly frequency and duration. Test-retest reliability was acceptable (ICCs 0.56-0.70).

*Single Item Global Quality of Life Scale (SIG-QOL)* [55] is a single item question measuring quality of life using a visual analogue scale ranging from 0 to 100. The single-item scale has high test-retest reliability (ICC was 0.87) and has shown high correlations with multi-item scales.

*Health-Promoting Lifestyle Profile II* (HPLP II) [56] is a 52-item questionnaire composed of six subscales including health responsibility, nutrition, physical activity, stress management, interpersonal relations, and spiritual growth. The English version of HPLP-II has shown high internal consistency and test-retest reliability.

*Global Sleep Assessment Questionnaire* (GSAQ) [57] is 11 items covering mood, life activities, and medical issues as they relate to sleep and symptoms associated with disorders of sleep. The GSAQ test-retest reliability reported the ICC ranged from 0.51 to 0.92.

#### Aim 2: psychological wellbeing

*General and Memory Specific Control Beliefs Scale* [58] measures perceived control over cognitive health. The scale is composed of two sets of items focusing on general and memory-specific control beliefs.

*Philadelphia Geriatric Center Morale Scale* (PGCMS) [59] is a 17-item scale measuring dimensions of emotional adjustments in persons aged 70 to 90. It provides a multidimensional approach to assessing the state of psychological wellbeing and perceived morale using three factors: agitation, attitude toward own aging, and loneliness dissatisfaction. Test-retest reliability ranged from 0.91 after five weeks to 0.75 after three months.

The *NIH Toolbox Meaning and Purpose Short Form* [60] is an 8-item, form that assesses the degree to which participants feel their lives matter/make sense. Each item is rated on a 5-point Likert scale with responses ranging from “strongly disagree” to “strongly agree” and from “not at all” to “very much.” The internal consistency is high (Cronbach alpha was 0.92).

*Patient Health Questionnaire-9* (PHQ-9) [61] is a reliable and valid (in a variety of patient populations) multipurpose instrument for screening, diagnosing, monitoring, and measuring the severity of depression.

*Generalized Anxiety Disorder 7-item (GAD-7) Scale* [62] is a commonly used measure of anxiety with 7 total items with high internal consistency (Cronbach alpha was 0.92) and test-retest reliability (ICC was 0.83).

*General Self-Efficacy Scale* (GSE) [63] is a 10-item psychometric scale that assesses optimistic self-beliefs to cope with a variety of difficult demands in life. Empirical evidence suggests that the GSE is a reliable and valid unidimensional measure across different cultural contexts.

#### Aim 3: cognition

*Multifactorial Memory Questionnaire* (MMQ) [64] is a measure constructed to reflect aspects of memory that are potentially amenable to clinical intervention. The Contentment subscale contains 18 statements that assess emotions and perceptions about current memory ability including anxiety, embarrassment, and irritability. The Ability subscale contains 20 items phrased as memory failures in everyday memory situations (e.g., forgetting an appointment). The Strategy subscale measures self-reported cognitive strategy use. The MMQ has been shown to have adequate content validity, test-retest reliability (0.86-0.93), internal consistency (0.83-0.95), and convergent and discriminant validity.

*Montreal Cognitive Assessment* (MoCA) [65] is a screening instrument that assesses multiple cognitive domains with total score ranging from 0 to 30 points, and a cut score of 24 has demonstrated very good specificity (by correctly identifying 87% of healthy participants) and excellent sensitivity when differentiating Mild Cognitive Impairment (90%) and Alzheimer disease (100%) from healthy comparisons. Test-retest reliability (patients tested 35 days apart) was high, with an intraclass correlation coefficient of 0.92. The internal consistency was also found to be high (Cronbach alpha on standardized items = 0.83).

### Data collection and plan

Research staff will be trained by the PI and responsible for all baseline and follow-up evaluations. At each assessment, staff will schedule the next assessment, check current contact information, and get updates on the Veterans’ status to promote retention. Research staff are responsible for entering all data into VA Research Electronic Data Capture (REDCap), a HIPAA-secure, web-based data collection and management platform managed by the VA. The PI will perform monthly audits on the database beginning after baseline data entry to ensure that data entry timelines are on target. Drs. Tripodis and Frank will only have access to de-identified data. Drs. Tripodis and Frank will assist with analysis after data collection and at each follow-up time point. Drs. O’Connor and Moo will meet monthly with Drs. Tripodis and Frank to review data as it is analyzed.

The statistical analyses will be broken down by study aim. For study aims 1 through 3, linear mixed regression models will be used to test the effect of AgeWISE-AP. The models will include linear effects of time with random intercepts and slopes for time. The dependent variables will include baseline measures and all the scores in all the follow-up visits. The primary models, run in all participants, will also include the following predictors: baseline age, sex, education (years), time (years from baseline), and the interaction (cross-product) of each predictor with time. For these models, the intervention x time interactions, reflecting differences in slopes between the intervention and the control group will be of primary interest. We do not expect any missingness in baseline covariates, and imputations will not be performed for longitudinal models since linear mixed effects models assume missingness at random. Factor analyses will be conducted to assess the validity of our approach (i.e., measurement structure and invariance). For power calculations, we used a simulation-based approach with an alpha of 5%, and an assumed participant dropout rate of 15% over the follow-up period. Our proposed sample size of 64 participants per group (128 total) will allow us to detect at least an 8% group difference in all our main outcomes over 6 months, with at least 80% power.

For exploratory *Aim 4*, we will acquire structural imaging data at baseline and one year on a subset (15 per group) of participants to determine volumetric changes in regions of interest. Neuroimaging data will be acquired on a 3 T Siemens Magnetom Prismafit scanner (Trio-upgrade). Prismafit data will be collected with a 20‐channel head coil. Two‐high resolution whole‐brain T1‐weighted images using Magnetization‐Prepared Rapid Gradient Echo (MP‐RAGE) volumes (TR/TE = 2.53 s/3.35 ms, flip angle = 7°, 1 mm isotropic) will be acquired. Scans will be 3D sagittal acquisitions with 176 contiguous slices (imaging matrix = 256 × 176, in‐plane resolution = 1 mm, slice thickness = 1 mm). Vertex‐wise general linear models will be performed using FreeSurfer to examine longitudinal changes in regional cortical thickness and volume throughout the cerebral cortex and subcortical gray and white matter in the intervention and control groups.

#### Study timeline

The proposed study timeline is outlined in Table 2. The first 3 months of the project (Year 1, Q1) were dedicated to study start up activities, including staff hiring and training, designing recruitment materials, printing study data collection packets and AgeWISE group manuals, and developing the manual and workbook used by the Brain Health Interventionist. Recruitment and baseline data collection began in Year 1, Q2 at an anticipated rate of 1–2 Veterans a week, projected at approximately 16 Veterans a quarter. Veterans are randomly assigned to AgeWISE-AP or control (*n* = 8 intervention, *n* = 8 control each quarter). The AgeWISE-AP intervention (20-weeks total), consisting of 12 weeks of the AgeWISE group and 8 sessions (held over 12 weeks) of the individualized AP, will began in Year 1, Q3, with months 7–9 (Q3) engagement in the first AgeWISE group and months 9–11 (Q3, Q4) engagement in the first AP. Approximately 8 AgeWISE-AP intervention groups will run in total, beginning each quarter thereafter (Y1 Q4 through Y3 Q2) comprising approximately 7–8 Veterans each for a total of 64 intervention Veterans, meeting the recruitment goals based on power calculations. Three-month follow-up data collection will begin in Year 2, Q1, and 6-month follow-up data collection will begin in Year 2, Q2. All data will be collected by Year 4, Q1, month 3. Year 4 Q1 and Q2 will be devoted to final data analysis, manuscript preparation, and submission, with Year 4 dedicated to grant preparation to further develop AgeWISE-AP with the goal of establishing mechanisms for sustained intervention adherence. We will seek funding opportunities looking to examine the effectiveness of rehabilitation interventions that include Veteran self-management components and/or clinician-directed adjunct approaches that allow for longer term monitoring of adherence and functional outcomes in participants.

## Discussion

### Study implications

As described above, age is the number one risk factor for cognitive decline and dementia. One way to reduce dementia risk is by engaging in lifestyle interventions. Multi-component interventions that provide brain aging education, improve feelings of control over brain aging, and deliver practical assistance with creation of an executable action plan are critically needed to improve the brain health of aging Veterans to reduce risk for cognitive decline. The AgeWISE-AP program aims to directly target and improve older Veterans’ engagement in brain healthy lifestyle activities, psychological wellbeing, and cognition.

### Dissemination plan

The current project will result in a revised manualized AgeWISE group protocol for participants and leaders. The project will also result in a manualized protocol for the individual Brain Health Interventionist sessions, along with an individual participant workbook. These assets will be used in a subsequent submission for multi-site funding to disseminate the program. If effective, the AgeWISE-AP program has promising scalability, with the role of Brain Health Interventionist easily adaptable and customizable to other VA facilities along with the use of the manualized materials. We intend to disseminate findings from the current study through conference presentation and publication in peer-reviewed journals.

### Potential study limitations

The AgeWISE-AP program is a 12-week group commitment, with 8 additional weeks of individualized work, for a total of 20-weeks of programming. A limitation to the study may be the high level of motivation of AgeWISE-AP participants, which may bias the results and reduce generalizability to less motivated individuals. The program is also run within the VA system of care and, as such, may not be fully generalizable to settings outside VA. In addition, we expect that the VA cohort we are recruiting from will be largely male. We will make every effort to recruit female participants.

### Adverse events

While we do not anticipate adverse events, it is possible that during the individual sessions, when participants are asked to describe their background and set personalized goals, that they may bring up issues that are upsetting to them. For any participant that appears to be struggling with anxiety or depression, or voices a need to discuss emotionally upsetting events, we will offer the option to discuss these additional concerns with a mental health professional at the VA facility. Also, as is true in all group settings, we are not able to fully guarantee confidentiality among members participating in AgeWISE during group sessions. To address this, group participants are made aware of the potential limits to confidentiality during the first session and we ask all group members to agree to keep anything discussed in the group private, acknowledging that we have no way to ensure group members do so.

## Data Availability

No datasets were generated or analysed during the current study.

## Abbreviations

AgeWISE: Aging Well through Interaction and Scientific Education program
AgeWISE-AP: Aging Well through Interaction and Scientific Education program-Action Plan
CBOC: Community Based Outpatient Clinic
CHAMPS: Community Healthy Activities Model Program for Seniors Questionnaire
cIRB: Central Institutional Review Board
DRIVE: Durability of Rehabilitation Interventions for Veterans
GSAQ: Global Sleep Assessment Questionnaire
GSE: Generalized Self-Efficacy Scale
HIPPA: Health Insurance Portability and Accountability Act
HPLP-II: Health-Promoting Lifestyle Profile II
ICC: Intraclass Correlation
MeDi: Mediterranean Diet
MMQ: Multifactorial Memory Questionnaire
MoCA: Montreal Cognitive Assessment
MP-RAGE: Magnetization‐Prepared Rapid Gradient Echo
REDCap: Research Electronic Data Capture
RR&D: Office of Rehabilitation Research and Development
PGCMS: Philadelphia Geriatric Center Morale Scale
PHQ-9: Patient Health Questionnaire-9
PI: Principal Investigator
PTSD: Post-Traumatic Stress Disorder
VA: Department of Veterans Affairs
VAMC: VA Medical Center
